# Biostimulant Effects of Rich Mannuronate-Alginate and Their Thermic-Acidic Depolymerized Derivates on *Triticum aestivum*

**DOI:** 10.3390/polym17243261

**Published:** 2025-12-08

**Authors:** Aldo Borjas, Fatima-Zahra Ahchouch, Niniva Ghosh, Surya Rajasekaran, Céline Dupuits, Said Mouzeyar, Redouan El Boutachfaiti, Emmanuel Petit, Roland Molinié, Cédric Delattre, Jane Roche

**Affiliations:** 1 Génétique, Diversité et Ecophysiologie des Céréales (GDEC), Université Clermont Auvergne, INRAe, 63000 Clermont-Ferrand, France; aldo.borjas@doctorant.uca.fr (A.B.); fatima_zahra.ahchouch@doctorant.uca.fr (F.-Z.A.); jane.roche@uca.fr (J.R.); 2 Institut Pascal (IP), Clermont Auvergne INP, Université Clermont Auvergne, CNRS, 63000 Clermont-Ferrand, France; 3 UMRT INRAe 1158 BioEcoAgro-BIOPI, IUT-GB, Université de Picardie Jules Verne, 80025 Amiens, France; 4 Institut Universitaire de France (IUF), 75005 Paris, France

**Keywords:** alginate, depolymerization, wheat, biostimulation, hydroponic culture, ascorbate/dehydroascorbate

## Abstract

Wheat (*Triticum aestivum* L.) productivity is frequently compromised by environmental stressors such as drought, salinity, and nutrient deficiencies. The application of biostimulants has emerged as a promising strategy to mitigate these challenges by enhancing plant growth, resilience, and nutrient uptake. This study investigates the biostimulant effects of alginate gels and their depolymerized derivatives, particularly alginate oligosaccharides (AOS), on wheat seedling development. Treatments were applied in hydroponic systems at 50 and 500 mg/L to assess morphological and physiological responses. Depolymerized alginates, especially AOS at 500 mg/L, enhanced root length, compared to control by 40% (Kruskal–Wallis; *p*.adj ≤ 0.001). Smaller oligomers (<2 kDa) showed better biological activity compared to larger molecules (Mw > 2 kDa). Indeed, AOS application modulated oxidative stress by improving the ascorbate/dehydroascorbate (AsA/DHA) ratio as the control, indicating a stronger antioxidant response and a reduced ROS accumulation without stress. These findings suggest that AOS not only promotes root and shoot growth but also enhances the plant’s endogenous ability to mitigate oxidative damage. The dual action, growth promotion and stress tolerance, position AOS as a promising biostimulant for improving wheat productivity in environmentally challenging conditions.

## 1. Introduction

Wheat (*Triticum aestivum* L.) is one of the most important cereal crops worldwide, providing essential nutrients for human and animal consumption. However, environmental stresses such as drought, salinity and nutrient deficiency significantly impact wheat productivity. In this context, the application of biostimulants has gained attention as an innovative strategy to enhance plant growth, yield, and resilience.

Biostimulants are defined as substances and microorganisms that, when applied to plants in poor soils, stimulate natural processes to enhance nutrient uptake, nutrient efficiency, tolerance to abiotic stress, and crop quality [1]. These products include humic substances, protein hydrolysates, seaweed extracts, microbial inoculants, and polysaccharides like alginates and chitosans. Biostimulants act by activating plant defense mechanisms, improving nutrient uptake, and enhancing stress tolerance through metabolic and structural changes. Carbohydrate-based biostimulants are recognized for their ability to elicit plant responses like those triggered by natural stress signals [2]. These compounds trigger the cascade of signaling molecules, modulating hormone balance, antioxidant activity, and osmotic regulation.

Alginates extracted from brown algae are composed of hetero/homo-blocks of mannuronic (M) and guluronic (G) acids. The biological activities of alginates are correlated with the DP (Degree of Polymerization) and their M/G ratio. It has been reported that the mannuronic fraction of alginates is responsible of their biological activity. Therefore, the evaluation of active molecules must consider the comparisons of the size and the ratio of M/G or at least the specification of these two main elements. With low DP, oligoalginates (AOS) have been shown to increase seed germination, seedling and root elongation and to induce resistance against pathogens [3]. As elicitors, they trigger signaling mechanisms via ROS and the activation of plant defense system, PR-Protein synthesis, polyphenol oxidase (PPO) and phenyl alanine lyase (PAL), and the enhancement of antioxidant systems like POD and CAT [4,5,6].

The bioactivity of alginate oligosaccharides (AOS) has been widely studied for their capacity to enhance growth and stress tolerance in cereals such as *Triticum aestivum*. A mixture of AOS (DP 2–6) applied as foliar spray significantly improved wheat biomass and relative water content (RWC) under both normal and osmotic stress (PEG 15%) [7]. These treatments also activated malondialdehyde (MDA) content and increased proline accumulation, indicating enhance osmoprotection. At the molecular level, AOS triggered the upregulation of drought-responsive genes linked to the abscisic acid (ABA) signaling pathway, including *LEA1*, *psbA*, *SnRK2*, and *PCS*, suggesting crosstalk between AOS perception and ABA-mediated stress responses.

Another major mechanism involves nitric oxide (NO) signaling. NO acts as a signaling and protective molecule that mediates crosstalk among ABA, ethylene, and auxins, regulating root development, flowering, senescence, and seed germination under stress conditions [8,9]. AOS were shown to stimulate endogenous NO production in a dose-dependent manner, promoting root elongation in wheat at 10–40 mg/L [10]. Inhibition assays using nitrate reductase and nitric oxide synthase blockers confirmed that AOS induced root development and that this mechanism is mediated via NO generation. Similar observations were made in *Oryza sativa*, in which AOS applied at 10–80 mg/L enhanced root biomass by up to 30% at 20 mg/L, correlated with an increase in auxin levels and a decreased IAA-oxidase activity [11].

Because of their antioxidant promoting activity, AOS also confers resilience to oxidative stress caused by heavy metals. Under cadmium exposure, seed primming with AOS (1 g/L for 5 h) improved wheat tolerance by enhancing antioxidant enzyme activity, reducing MDA accumulation, and increasing chlorophyll content and seedling length [12]. Comparable effects were observed in *Zea mays*, where seed priming with AOS (7.5 g/L for 15 h) increased germination energy, seed viability, and shoot and root growth by 146% and 118%, respectively [13].

Field experiments confirmed that AOS improved wheat yield components in a dose dependent manner. Foliar application at 25, 50 and 10 mg/L during greening, jointing and booting stages showed that 25 mg/L was the most effective concentration, increasing spike number, grain number per spike, thousand kernel weight, and final yield by 3.2–17.5%. Higher doses (100 mg/L) reduced yield components, emphasizing the need for precise dosage optimization and consideration of multifactorial field variability [14].

Beyond oligosaccharides, polymeric alginate fractions and calcium-crosslinked derivates also exhibit biostimulants activity. Wheat seed primming with calcium alginate nanoparticles (~250 nm, 0.1 g/L) enhanced root (+69%) and shoot (+21%) growth, increased auxin content, upregulated *ARF* and *YUCCA9* gene expression involved in auxin signaling, and repressed UGT (IAA-oxidase), confirming their auxin-related biostimulant effect [15].

Given the broad spectrum of biological effects attributed to AOS, the production of depolymerized alginates has been extensively developed to convert raw alginates into low molecular weight alginates (LMW) and oligoalginates (AOS), which can be classified into physical, chemical and enzymatic methods [16]. The glycosidic bonds of alginates are sensible to cleavage by acidic/basic-hydrolysis, oxidative, enzymatic reactions, and can also be combined with physical methods as UV-light, gamma radiation, plasma, ultrasounds, mechanical milling or thermic process [17,18,19,20,21].

The enzymatic approach is the most used to obtain oligoalginates [22,23,24,25]; the alginate lyase allows the degradation of the alginate through beta-elimination mechanism, forming unsaturated residues at the non-reducing end (C4–C5) over the oligosaccharides produced. On another hand, the chemical depolymerization reported is mainly realized by acidic/basic hydrolysis of the glycosidic bond giving saturated oligosaccharides. As well, oxidative depolymerization using H_2_O_2_ has been widely used; the mechanism is reported through the formation of hydroxyl radical, leading to the scission of the glycosidic bond. Nevertheless, in harsh condition the pyranose ring can be open in a random scission pattern (carboxyl group at the C1) [26].

Concerning physical methods, ultrasound assisted depolymerization (59 kHz/35 °C/2–4 h) of alginate has been used [27], obtaining a medium molecular weight alginate without the generation of oligoalginates. In addition, the use of a double step method with both physical and chemical approaches, under effect of temperature/H_2_O_2_ and ultrasound has been reported [26]; the uses of Fe as a catalyst enhanced depolymerization at 70 °C/90 °C without ring-opening. The same yields were observed when the oxidative process through H_2_O_2_ was selected over the thermic-acidic depolymerization [28].

Combined methodologies were reported as more efficient [18,26,28,29]. Holme et al. elucidate the thermic-acidic process to obtain depolymerized alginates [29]. They observed a pH dependent process, correlating the concentration of protons to the DP. They found also a plateau phase from pH 5 to 8 as a low rate depolymerization; outside those pH ranges, the degradation rates increase, by means of acid hydrolysis (H^+^ catalysis at low pH) or by *β*-elimination (HO^−^ catalysis at high pH).

The present work aims to perform a first screening and to evaluate the biostimulant effects of alginates and the thermic-acid depolymerized products on wheat seedlings under a hydroponic system, focusing on their influence on root and shoot development, biomass production, and physiological responses. Different molecular weights and concentrations of alginates have been tested to identify the best formulation for wheat biostimulation.

## 2. Materials and Methods

### 2.1. Alginates and Thermic-Acidic Depolymerized Alginates Preparation

Three types of alginates purchased from Algaia (Lannilis, France) were tested as raw materials: rich guluronic acid alginate (M/G 0.7), rich mannuronic acid alginate (M/G 1.4) and a calcium reticulated alginate (Ca-Al).

The thermic-acidic depolymerization was realized on the high rich mannuronic acid content alginate (M/G) supplied by Algaia (Lannilis, France), based on the procedure established by Mellal et al. [30] with some modifications to produce a range of degradation products. Alginate solution (20 g/L) was prepared on deionized water, and the pH was adjusted with NaOH 1 M at 4.4 followed by an autoclave-cycle (40 min; 120 °C; 1 bar). After cooling, the oligoalginates of low molecular weight were obtained by filtration. This step was performed on Vivaflow200 system with a PES membrane of 3000 MWCO (Sartorius Stedim Biotech GmbH, Gottingen, Germany). The autoclave cycle and the filtration steps were performed ten times sequentially. A retentate sample of 20 mL was taken after each cycle.

### 2.2. Physico-Chemical Analysis

#### 2.2.1. Sec-MALLS of Alginates and Its Derivates

Size exclusion chromatography (SEC) coupled with multi-angle laser light scattering (MALS) was used for molecular weight determination of alginate compounds. The chromatographic set-up used consists of an isocratic pump (LC-20AD, Shimadzu, Duisburg, Germany), a column (SB-G guard column and three columns in series SB-806 HQ, SB-804 HQ, and SB-803 HQ, 300 mm L × 8 mm I.D., Shodex Showa Denko K.K., Tokyo, Japan) and following by the MALLS detector (MiniDAWN TREOS II, Wyatt Technology Corporation, Santa Barbara, CA, USA) and a refractive index detector (RID-10 A, Shimadzu, Duisburg, Germany).

The system was eluted with NaNO_3_, 0.1 M, and NaN_3_, 2.5 mM, at a volume flow of 0.5 mL/min. Alginate and its depolymerized derivates (2.5 to 20 mg.mL^−1^) were filtered through a 0.45 µm membrane filter (Grace Altech, Columbia, SC, USA) and were injected through a 100 µL full loop. Data acquisition and processing were performed using ASTRA 7.2.2 software. Specific refractive index increments (dn/dc) of 0.150 were used according to the literature.

#### 2.2.2. ^1^H NMR Spectroscopy Analysis for Guluronic and Mannuronic Fractions on Alginate and Its Depolymerized Derivates

The freeze-dried polymeric alginates were dissolved in D_2_O at 15 g/L. The ^1^H NMR spectrum was recorded at 80 °C on a Bruker Avance 500 MHz spectrometer (Bruker, Billerica, MA, USA) operating at 500.08 MHz for ^1^H, using a multinuclear probe BBI 5 mm. A classical 1D proton was acquired. The sequence repeat was D1-90°-AQ, where D1 (20 s) is the relaxation delay, 90° is the already determined 90° radio-frequency pulse length and AQ (9.36 s) is the data acquisition time. The spectrum was acquired using eight scans of 128 K data points, using spectral widths of 7002.8 Hz. The freeze-dried samples of depolymerized alginates were dissolved in D_2_O at 15 g/L. ^1^H NMR spectrum was recorded at 15 °C on a Bruker Avance 600 MHz spectrometer operating 600.13 MHz for ^1^H equipped with a z-gradient inverse probe head (TXI, 5 mm tube). Classical 1D ^1^H-NMR spectra were collected using 128 scans and four dummy scans of 128 K data points and a spectral width of 8417 Hz with a relaxation delay of 20 s. The FID was multiplied by an exponential weighing function corresponding to a line broadening of 0.10 Hz prior to Fourier transformation.

All non-zero filled obtained spectra were manually phased, baseline corrected, and calibrated to TMSP (TriMethyl Silyl propionate) at 0 ppm, all using TopSpin 3.6 (Bruker BioSpin, Ettlingen, Germany).

The signals obtained in ^1^H NMR spectra were used to determine the distribution of mannuronic (F_M_) and guluronic (F_G_) fractions, as well as the frequency of heterogeneous blocks (F_MG_ or F_GM_) and homogenous blocks (F_GG_ and F_MM_) [31].

The distributions of GulA and ManA in alginates (F_G_ and F_M_), and the homogenous (F_GG_ and F_MM_) blocks of alginate were calculated after analysis of ^1^H spectra signals at 4.95–5.20 (A_I_) for the guluronic acid anomeric proton, signals at 4.6 and 4.8 ppm (A_II_) for the overlap between the mannuronic acid anomeric proton (M-1) and the C-5 of alternating blocks (GM-5) and the signal at 4.4 and 4.5 ppm (A_III_) for the guluronic acid H-5 (GG-5G) according to the method described by Grasdalen [31] using the following Equations (1)–(5)

(1) $$F_{G}=\frac{A_{I}}{A_{II}+A_{III}}$$

(2) $$F_{GG}=\frac{A_{III}}{A_{II}+A_{III}}$$

(3) $$F_{M}=1-F_{G}$$

(4) $$F_{MM}=F_{M}-F_{MG}$$

(5) $$\frac{M}{G}=\frac{F_{M}}{F_{G}}=\frac{1-F_{G}}{F_{G}}$$

### 2.3. Plant Biostimulation Assays

#### 2.3.1. Experimental Device in Hydroponic System

Seven days old and fifteen days old winter wheat (*Triticum aestivum*) seedlings were used to evaluate the different treatments. The sterilization of wheat seeds was performed by soaking seeds in 3% bleach for 10 min, followed by three washing steps with sterile ultra-pure water. Seed germination was performed by immersing the seeds in sterile ultra-pure water overnight in the growth chamber followed by stratification, where seeds were transferred on to a sterile, moist, tissue paper in a Petri plate and stored at 4 °C for 48 h. Sixteen sterile seeds were sown onto each grid floating on a polystyrene piece in a hydroponic device containing 130 mL of half Murashige and Skoog medium (MS ½ media) at pH 5.7; for each treatment, four hydroponic systems were sowed (64 seedling per treatment). Hydroponic devices were placed in a growth chamber at 21 °C/60% RH with a photoperiod of 16/8 h/400 µmol/m^2^/s.

#### 2.3.2. Raw Materials Biological Activity Screening

To identify the alginate-based treatment with a high biological activity, two alginates with different M/G ratios (Al M/G 1.4 and Al M/G 0.7) and a calcium-alginate (Ca Al) supplied by the company Algaia were tested and compared with a control without treatment (only MS media; four hydroponic system per treatment). After 7 days of growth, the treatments were applied at 1 g/L in MS medium, following the same concentration chosen in the previous work performed by Bouissil et al. [3]. The seedlings were harvested after 7 days of treatment (15 days of growth). The seedling development and plant immune molecular markers were evaluated as further described.

#### 2.3.3. Immunostimulation Markers Screening Evaluation

Following the treatment, the tissues were sampled separately and ground in liquid nitrogen. The RNAs from these tissues, used for further analysis, were extracted according to the Macherey Nagel RNA Plant and Fungi kits (Macherey Nagel, Düren, Germany) (740,120.250) for the roots. The concentration of RNA obtained was determined by Nanodrop oneC (Thermo scientific, Waltham, MA, USA) and the quality determined by electrophoresis. Two μg of RNAs were retranscripted using Maxima H minus first strand cDNA synthesis kit with dsDNAse (Thermofisher K1682) according to the instructions provided (DNAse: 2 min 37 °C, Rt: 30 min 50 °C, 5 min 85 °C). The samples were stored at −80 °C for RNAs and −20 °C for cDNAs.

Primers provided by Eurofins genomics (Table A2; Appendix A), specific to our genes of interest and corresponding for the three wheat genomes (ABD) were designed by sequence alignment. The Rt-qPCRs were carried out from 1/20 diluted cDNA on the Gentyane genotyping platform in Clermont-Ferrand (https://gentyane.clermont.inrae.fr/, accessed on 16 August 2025). A Hamilton Genomics Starlet robot was used for the pipetting of the cDNAs as well as the Green 1 Master mix (Roche 04887352001) used to reduce technical variability. The Rt_qPCR conditions applied by the use of a Roche 480 Light Cycler (Roche, Basel, Switzerland) correspond to a pre-incubation phase (95 °C 10 min), 45 amplification cycles (95 °C 10 s, 60 °C 15 s, 72 °C 15 s) and a melting curve phase (95 °C 15 s, 60 °C 15 s, 95 °C continuous with acquisition every 5 °C). The results obtained using the LightCycler480SW1.5 software were normalized from our household gene (*TaTUB*) and then processed using the 2^−ΔΔCt^ method [32]. For each molecular analysis, four biological replicas were used.

#### 2.3.4. Biostimulant Effect of Depolymerized Products

Over the depolymerized products, 3 retentates were selected based on their molecular weight size to cover different ranges of sizes (R1, R3 and R10). These treatments were compared with the alginate with high ratio M/G and the mixture of oligoalginates (AOS) obtained after the ultrafiltration step.

Treatments were prepared at two concentrations, 50 mg/Land 500 mg/L, by dissolving the products in MS medium. Seven-day old wheat seedlings were used to evaluate the effect of alginates and AOS on their development. Treatments were applied on the hydroponic system and after 7 days of treatment, plants were harvested. The plant biomass and seedling development was evaluated as further described.

#### 2.3.5. Biomass Evaluation and Root/Shoot Measurements

For each hydroponic container, over the 16 seedlings, eight seedlings were sampled to quantify the biomass (32 seedlings per treatment). Shoots and roots were weighted separately using an analytical balance (Sartorius CP224s; Sartorius AG, Gôtitingen, Germany). The dry weight (DW) was obtained after drying the shoots and roots in an oven for 72 h at 70 °C. The remaining seedlings (32 seedlings per treatment) were harvested by separating roots and leaves which were promptly frozen in liquid nitrogen and stored at −80 °C for further analysis.

Roots and shoots were evaluated using ImageJ software (version 1.54d) using the method proposed by Lobet et al. [33] with the Smart-Root plug-in. Images were obtained using 6–8 seedling samples scanned using a scanner (Sharp MX3570NEU, Sharp, Osaka, Japan; color scan 600 DPI). The root length, diameter, surface and volume were extracted from the analysis generated by Smart-Root. The same procedure was used to calculate shoot length and leaf surface area.

#### 2.3.6. Antioxidant Activity of Depolymerized Alginate Fractions on Wheat

The quantification of ascorbate from leaves was carried out based on a detailed extraction and colorimetric assay protocol proposed by Gillespie et al. [34].

From the seedling harvested and frozen on liquid nitrogen, the leaves were ground using a Bead Ruptor 96 well plate homogenizer (Omni International, Kennesaw, GA, USA) and stored at −80 °C for further analysis.

A sample of 10 mg of plant material was collected and extracted with 2 mL of 6% (%*w*/*v*) Trichloroacetic acid (TCA) and vigorously agitated for 1 min, followed by a centrifugation step (13,000 g/5 min/4 °C) to separate cell debris. Then, the supernatant was carefully transferred to a new microtube and kept on ice to quantify immediately the reduced ascorbate and total ascorbate.

Assays for blanks, standards and samples were performed in quadruplicate for both reduced AsA (ascorbate) and total AsA. 100 µL of 75 mM phosphate buffer (PBS, pH 7) was mixed with 200 µL of either 6% (%*w*/*v*) TCA (blanks), AsA standards (0.2–2 mM AA) or sample extract into a 2 mL tube.

For Total AsA, 100 µL of freshly prepared 25 mM DTT was added to reduce any oxidized ascorbate. After 10 min of incubation at room temperature, 100 µL of 0.5% (%*w*/*v*) N-Ethylmaleimide (NEM) was added to inactivate DTT excess, followed by 30 s incubation. For AA assays, 200 µL of water were added instead of DTT and NEM.

The colorimetric reaction was started by the addition of 500 µL 10% (%*w*/*v*) TCA, 400 µL 43% H_3_PO_4_, 400 µL α-α’-bipyridyl and 200 µL 3% (%*w*/*v*) FeCl_3_ to all assay’s tubes. The tubes were agitated vigorously to avoid the formation of a white precipitate. Then the tubes were incubated (37 °C/1 h). After incubation, samples were read in spectrophotometer at 525 nm.

Based on the standard curve of AsA, the quantity in nmol of AsA were determined. Oxidized AsA (DHA) is calculated as the difference between the total pool and the reduced pool. As well the ratio AsA/DHA were calculated to obtain the redox state.

## 3. Results

### 3.1. Biological Activity Screening of Raw Alginates

#### 3.1.1. Physicochemical Characterization by Sec-MALLs and ^1^HNMR

The structures of both alginates show differences in molecular weight and monomers distribution (Table 1). Both alginates show high molecular weight: Al M/G 1.4 is an alginate of 281 kDa; meanwhile Al M/G 0.7 is an alginate of 234 kDa. Concerning the structure, Al M/G 1.4 is composed of 43% *α*-L-GulA (G) and 57% *β*-D-ManA (M) (M/G = 1.35) and differs from Al M/G = 0.7, which is composed of 60% *α*-L-GulA (G) and 40% *β*-D-ManA (M) (M/G = 0.67). Al M/G 1.4 shows the greater abundance of blocks MM compared with Al M/G 0.7 (0.54 > 0.12, respectively). For blocks GG, Al M/G = 1.4 has the greater abundance, followed by Al M/G = 0.7 (0.39 > 0.32, respectively).

#### 3.1.2. Biological Activity of Raw Alginates: Gene Expression Analysis and Physiological Analysis

**Figure 1:**
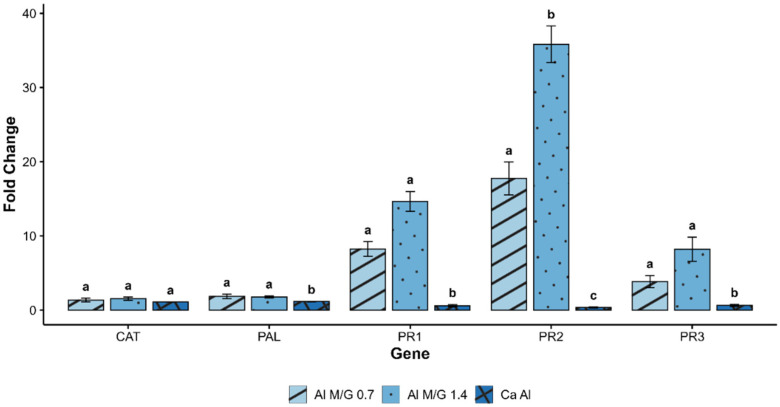
Fold change in gene expression level of *TaCAT*, *TaPAL*, *TaPR1*, *TaPR2* and *TaPR3* in seedlings treated with three different HMW alginates at 1 g/L: alginate with M/G = 0.7, alginate with M/G = 1.4 and calcium alginate. Gene expression was normalized with untreated seedlings. Values are represented as mean values ± SE. Different letters show significant differences. Statistical differences were assessed using Kruskal–Wallis test with Dunn’s post hoc and BH correction for multiple comparison (*p*.adj ≤ 0.05).

The expression level on five defense-related genes (*TaCAT*, *TaPAL*, *TaPR1*, *TaPR2* and *TaPR3*) were assessed in response to treatments with alginates differing in their M/G ratio (0.7 and 1.4) as well as calcium alginate (Figure 1). Compared with control (untreated), the expression of CAT and PAL remained largely unchanged across all treatments, suggesting limited involvement of these enzymes under the tested conditions. In contrast, significant up-regulation was observed for three genes: *TaPR1*, *TaPR3*, and particularly *TaPR2*, with the highest fold changes observed for the alginate samples with an M/G ratio of 1.4 (fold change of 36 for M/G 1.4, 18 for M/G 0.7 and 0.35 for Ca Al).

After 7 days of treatment with raw alginates at 1 g/L, significant differences were observed compared to the control (Table 2). Root length was strongly enhanced by the alginate with an M/G of 1.4, reaching ~16 cm (corresponding to +170% compared to the control), which was significantly higher than all of the other treatments (*p* < 0.05). But both of the alginates with M/G = 0.7 and calcium alginate also promoted root growth (~9.5 and ~8.5 cm, respectively; corresponding to +58% and +44%, respectively), even though their effects were not significantly different from each other. In contrast, shoot length was only enhanced by the alginate M/G 1.4 (~31 cm, corresponding to +64% compared to the control) while the other treatments showed similar shoot elongation (~18 cm).

### 3.2. Alginates Depolymerization and the Effect on Seedlings Development

#### 3.2.1. Molecular Mass and Structural Characterization of the Depolymerized Alginates Obtained by Thermic-Acidic Depolymerization

Thermic-acidic depolymerization results in a gradual reduction in molecular weight and uronic acid content with each cycle, as smaller fragments are progressively generated (Table 3). The polydispersity index (PDI) remains relatively stable throughout the reaction, suggesting that the depolymerization mechanism does not significantly affect the uniformity of the molecular weight distribution. The starting molecular weight (Mw) of the alginate is 281 kDa, with a PDI of 2.52 and 1606 uronic acid units. The AOS represents the mixture of filtrate fractions recovered after each depolymerization-ultrafiltration cycle. As expected, the filtration steps lead to the separation of low molecular weight oligosaccharides families (722 Da–380 Da). The PDI was maintained stable and low after each cycle 1.27–1.041. In addition, the Retentate (ROA-x) represents the higher molecular weight fraction of alginate retained during filtration. The molecular weight decreases significantly over cycles, from 11 kDa (cycle 1) to 1.97 kDa (cycle 10) and the PDI reduces from 2.26 (cycle 1) to 1.238 (cycle 10), indicating less molecular weight variation after the later cycles. The trend in uronic acid units shows a steep decline, dropping from 63 in cycle 1 to 11 in cycle 10, indicating significant degradation of uronic acid-rich fractions. These variations were used to select the samples tailored to specific requirements based on uronic acid molecular weight: R1, with 63 uronic acid units, represents the highest concentration of these units; R3, with 28 uronic acid units, reflects a medium range; and R10, with 11 uronic acid units, corresponding to the lowest molecular weight obtained in the retentate.

The mass profile of the filtrate yield 33% of oligosaccharide production (Table 4) over the 10 cycles, and the rest corresponding to the retentate with small alginates molecules around DP 11 (Table 3; R-10, Mw 1970 Da and 1.24 PDI).

**Figure 2:**
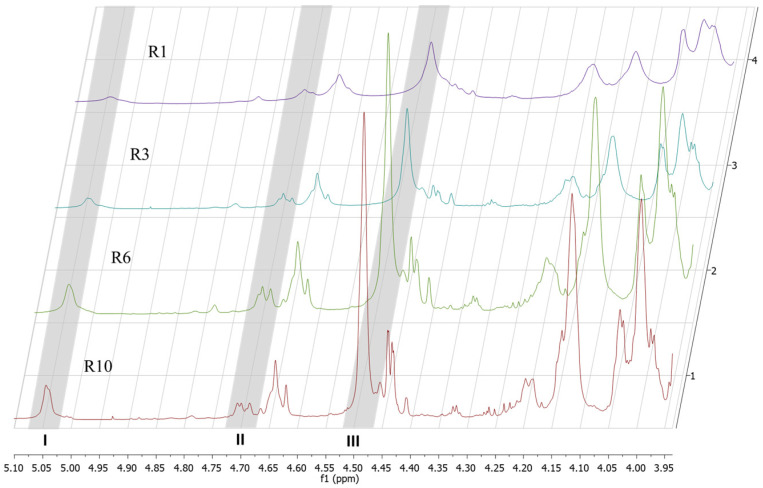
^1^H-NMR spectra of depolymerized alginates retentates (R1, R3, R6 and R10) and the three-signal used to calculate guluronic, and mannuronic-acid-blocks (A_I_, A_II_ and A_III_).

The structures of the retentates analyzed by ^1^H-NMR spectroscopy show how the thermic-acidic depolymerization process was performed (Figure 2 and Table 5). Over the autoclave cycles and filtration process, the percentage of α-L-GulA (G) increased from the initial Al M/G 1.4 to the last retentate (ROA 10), from 43% to 78%, respectively; in contrast, the β-D-ManA (M) content decreased in the retentate over the progression of the cycles, from 57% to 22%. Concerning the blocks MM, the process disrupted the homoblocks of β-D-ManA (MM) from 54% to 4%.

#### 3.2.2. Biostimulant Effects of Depolymerized Fractions

**Figure 3:**
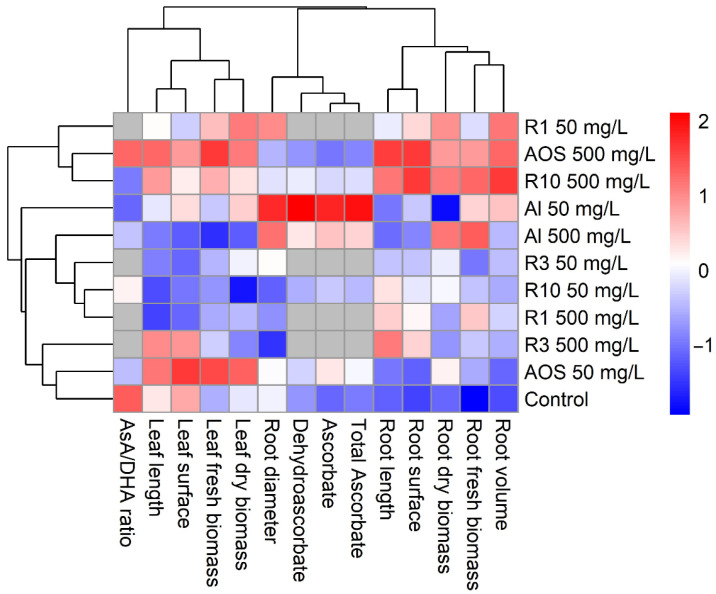
Heatmap of Z-score normalized mean values of morphological and redox-related parameter measured in wheat seedlings subjected to different alginates fractions. Treatments include the polymeric alginate (Al M/G 1.4), three retentates selected to cover the range of sizes, 63 units of UA (R1), 28 units of UA (R3), 11 units of UA (R10) and the AOS, applied at two concentrations, 50 and 500 mg/L, and compared to control. Rows represent the treatments, and columns represent the measured parameters: root and leaf fresh and dry biomass (FWR, DWR, FWL and DWL), root architectural traits (length, surface area, volume and diameter), leaf morphological traits (length and surface), and redox-related indicators (AsA, DHA, AsA/DHA and total AsA). Data were standardized by parameter using Z-score transformation. Red colors indicate higher-than-average values; blue colors indicate lower-than-average values. Gray cells represent missing data (NA). Both rows and columns were clustered using hierarchical clustering based on Euclidean distance and complete linkage.

The heatmap (Figure 3) reveals clearly a treatment-dependent multivariate shift. Hierarchical clustering splits the treatments into two main groups: a cluster including the mixture of AOS 500 mg/L, R10 500 mg/L and to a lesser extent, R1 50 mg/L, that showed above-average Z-scores across several traits, most notably in the ascorbate pool (AsA, total AsA) and selected biomass metrics; and a cluster containing AOS 50 mg/L, R3 500 mg/L, and the control, characterized by below-average root biomass/volume and lower redox values.

Variable clustering (hierarchical clustering based on Euclidean distance and complete linkage) grouped traits into coherent modules: root biomass (volume, fresh/dry biomass), root architecture (length/surface, diameter separate), leaf morphology (length/surface, fresh/dry biomass), and redox markers (AsA, DHA, AsA/DHA, total AsA). Overall, higher doses of AOS and R10 seemed to enhance redox status and growth-related traits, whereas R3 at 500 mg/L were neutral/negative across the different parameters measured.

##### Root and Shoots Biomass

In Table 6, most of the treatments show a tendency in biomass increase on the root and leaf tissues. Nevertheless, no significant difference was detected between the treatment and the control. This outcome is likely explained by the high intra-treatment variability, which was dependent on dose/size (Table A1); the AOS mixture at 500 mg/L shows robust effect over the different samples treated (~+30% ± 3.3 on roots dry weight and ~+20% ± 5.5 on leaves dry weight).

##### Root Morphology

**Figure 4:**
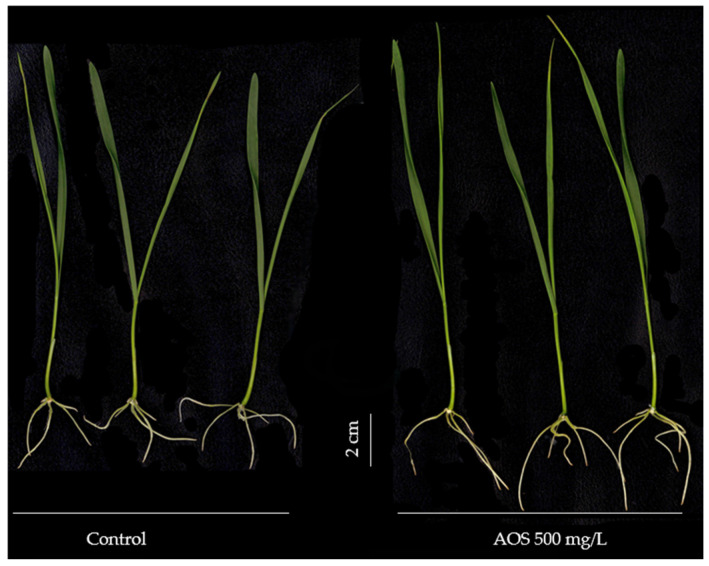
Wheat seedlings (seven days old) comparison between control and AOS 500 mg/L.

**Figure 5:**
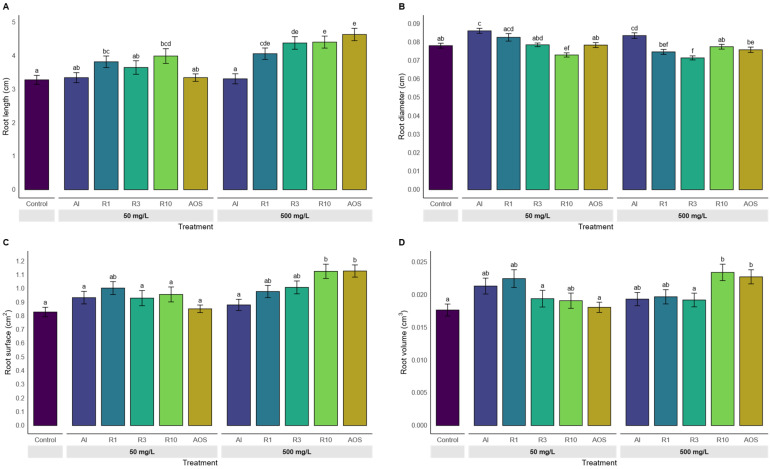
Effect of treatments on root morphology after 7 days of growth. (**A**) Root length, (**B**) Root diameter, (**C**) Root surface, and (**D**) Root volume of seedlings treated with alginate and its depolymerized derivates at 50 or 500 mg/L. Treatments include alginate HMW (Al), depolymerized alginate fractions (Rx_cycles), retentate 1, 3 and 10, and the oligoalginates mixture (AOS). Bars represent mean values ± SE. Different letters above bars indicate significant differences between treatments (Kruskal–Wallis test followed by Dunn’s post hoc test with BH correction, *p*.adj ≤ 0.05).

The root morphology (Figure 5) varied with both treatment and dose. Root length (Figure 5A) increased in a dose-dependent manner, among the depolymerized alginate fractions, R3, R10 and AOS induced the highest values at 500 mg/L (Figure 4; Comparison between Control and AOS 500 mg/L), significantly exceeding the control, the polymeric alginate (Al) and R1. In contrast, root diameter (Figure 5B) showed the opposite trend and was strongly treatment dependent, with Al yielding the largest diameters, significantly higher than the other treatments. Root surface area and root volume (Figure 5C,D) were also significantly increased by R10 and AOS to control, indicating enhanced root system development under these conditions.

##### Shoot Morphology

**Figure 6:**
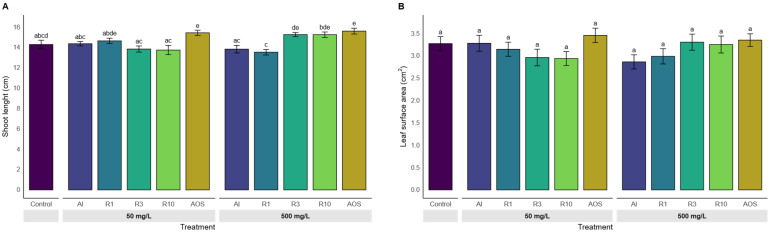
Effect of treatments on shoot morphology after 7 days of growth. (**A**) Shoot length, (**B**) Projected leaf surface area of seedlings treated with alginate and its depolymerized derivates at 50 or 500 mg/L. Treatments include alginate HMW (Al), depolymerized alginate fractions (Rx_cycles), retentate 1, 3 and 10, and the oligoalginates mixture (AOS). Bars represent mean values ± SE. Different letters above bars indicate significant differences between treatments (Kruskal–Wallis test followed by Dunn’s post hoc test with BH correction, *p*.adj ≤ 0.05).

Shoot length (Figure 6A) was significantly increased by AOS at both 50 and 500 mg/L. A dose-dependent effect was observed for R3 and R10, with the significantly highest shoot length at 500 mg/L compared to control. In contrast, the projected leaf surface did not differ significantly among treatments.

##### Shoot Redox State

**Figure 7:**
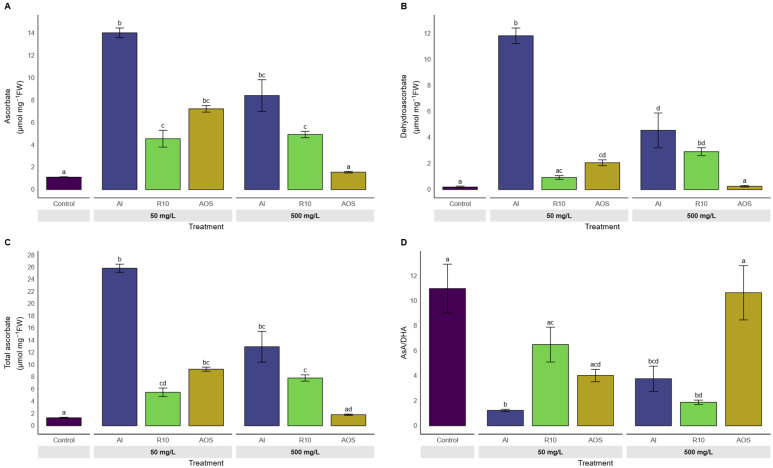
Effect of treatments on the redox state of leaves (ascorbate) after 7 days of growth. (**A**) Ascorbate, (**B**) Dehydroascorbate, (**C**) Total ascorbate, and (**D**) AsA/DHA ratio of seedlings treated with alginate and its depolymerized derivates at 50 or 500 mg/L. Treatments include alginate HMW (Al), depolymerized alginate fractions Retentate 10 (R10), and the oligoalginates mixture (AOS). Bars represent mean values ± SE. Different letters above bars indicate significant differences between treatments (Kruskal–Wallis test followed by Dunn’s post hoc test with BH correction, *p*.adj ≤ 0.05).

Figure 7 shows leaf redox status. Reduced ascorbate (AsA; Figure 7A) was highest under Al 50 mg/L, clearly above the control and the other treatments. R10 and AOS at 50 mg/L produced moderate increases compared to control, whereas at 500 mg/L AsA decreases for Al and AOS. Dehydroascorbate (DHA, Figure 7B) showed a marked rise with Al 50 mg/L, smaller increased with R10 and AOS at 50 mg/L, and very low values for AOS 500 mg/L. Consistently, the AsA/DHA ratio (Figure 7D) was the lowest under Al 50 mg/L, intermediate for R10 50 mg/L, and the highest under AOS 500 mg/L, similar to the control.

## 4. Discussion

The thermic-acidic depolymerization shows the decrease in the mannuronic fraction regarding the NMR results. This observation aligns with the findings of Holme et al. [29], describing that the blocks of mannuronic acid were more sensible to acidic hydrolysis when coupled to thermic processes compared to guluronic acid blocks. In our study, after the filtration step, the retentate NMR results show an augmentation of the guluronic acid fractions at each autoclave cycle; which suggests that the filtrate (AOS mixture) is rich in mannuronic acid.

Regarding the percentage of depolymerization (by means of the molecular weight distribution) the thermic-acidic process applied on the alginate leads to ~60% of depolymerization at the first cycle, beyond this cycle the percentage increased to 97% of depolymerization. Comparing with the oxidative process realized by Mao et al. [35], where they found 80% of depolymerization after 1 h of reaction and a stabilization of the degradation rate, the thermic-acidic process leads to a faster depolymerization (after one cycle (approximately 40 min: 61% of depolymerization; and 97.5% after the second cycle). This can be correlated to the fact that the oxidative process has exhausted the entire H_2_O_2_ quantity at 1 h [35] while the thermic-acidic depolymerization can be catalyzed by protons coming from the acid but also from the autocatalytic ability of the carboxyl groups of the mannuronic and guluronic acids which act as proton donors [29]. Thus, the depolymerization reaction continues after the first burst of depolymerization (first autoclave cycle).

The physiological effects of alginates with a high mannuronic acid content (mannuronic-to-guluronic acid ratio (M/G > 1) on plant growth was already described [11,19,36,37], and showed a notable increase in the cell wall structure, signal transduction, and root development.

The structure of the two alginates used in this paper was elucidated identifying two types of alginates: one with a high mannuronic acid content (0.57) with a ratio M/G of 1.4, and the second one with a lower content of mannuronic acid (0.4) with a ratio M/G of 0.7. The alginate with a M/G ratio of 1.4 showed better growth and development activities when applied to roots compared to the one having a lower ratio.

The molecular response, evaluated by q-RTPCR (on root system), showed an important overexpression of genes encoding PR-proteins (15-, 35-, 8-fold for PR1, PR2 and PR3, respectively) when using alginates with M/G ratio of 1.4, compared to alginate with a 0.7 ratio, and calcium alginate highly reticulated (CaAl). PR-1 proteins are conserved among diverse plant species and recognized as a reliable marker for activation of hypersensitive response (HR)-mediated defense pathways or the establishment of salicylic acid (SA)-mediated disease resistance in diverse plant species [38]. PR-2 and PR-3 are genes encoding a *β*-1,3-glucanase and chitinase families, respectively, largely described as belonging to a wide gene family involved in the induction of defense mechanisms [39]. Notably, the PR2 gene is the most overexpressed one, which is correlated with the composition of alginates. Jiang et al. reported that this enzymes’ family belongs to the glycoside hydrolases (GH), known to be involved in the DAMP’s signaling linked to the presence of glucans’ structure [40]. Furthermore, the laminarin has been demonstrated as potential elicitor of the PR2 in tobacco cells [41], where the structure can be correlated to alginates (mannose for laminarin and mannuronic acid for alginates).

The evaluation of isolated constituents from an alginate extracted from the macroalgae *Lessonia vadosa* were tested on wheat seedlings [36], demonstrating the potential of plant stimulation by both guluronic and mannuronic acids in their polymeric forms. In our conditions, both fractions exhibit elicitor capacities, with the mannuronic fraction showing a stronger response of the phenylalanine ammonia-lyase (PAL) and total peroxidase activities. This observation aligns with the idea that alginates with an M/G greater than 1 might enhance stress signal transduction and growth-promoting activities. In addition, a recent study performed on barley [37], shows that the molecular weight and the M/G ratio have a significant impact on the biomass production and shoot length as well as the soluble sugar content in shoots. Globally, they demonstrated that the mannuronic rich-oligoalginates (DP3–4) have the stronger growth and development of cereals when compared with alginates rich in guluronic acid. More precisely, the size of alginate would be the major parameter, showing that oligomers have a better biostimulant activity (growth and antioxidant activity) whereas polymeric alginates might have an enhanced immunostimulant activity (by means of PAL). Regarding the thermic-acidic depolymerization method, our results showed that, in most of the cases, the smaller fractions (AOS mixture and R10) triggers better growth stimulation and antioxidant protection on wheat seedlings, and aligns with the previous findings on cereals [5,12,13,37]. For example, Yang et al. found that the maximum positive effect on plant growth was observed with two main oligomers (DP3 and DP12) [37] close to R10 (DP11) and AOS (DP3) in our conditions.

Regarding the redox status, the AsA/DHA ratio could be considered as an indicator of the stress level that could be perceived by the plant [42,43]. A ratio greater than 1 indicates that the ROS produced will be easily managed by the antioxidant system, whereas a ratio less than 1 could be considered as a redox depletion marker. Our findings highlight that alginate’s application at low concentrations enhances both reduced and oxidized ascorbate pools but shifts the balance toward oxidation, while smaller-sized oligosaccharides (AOS) maintain a more reduced redox state [44]. This finding is in accordance with the results obtained by Yang et al. [37] on barley, showing a significant modification of the non-enzymatic antioxidant balance (AsA/DHA) and an increase in the antioxidant enzymatic capacity, precisely the peroxidase, catalase and superoxide dismutase capacity after oligoalginates application with low molecular weight. In the same idea, Ma et al. [12] showed that under heavy metal stress (Cadmium) and AOS treatment, wheat seedlings exhibit an enhancement of the antioxidant enzymatic activities under stress conditions. Taken together, this suggests the potential role of AOS in mitigating oxidative stress by preserving ascorbate in its active, reduced form.

## 5. Conclusions

Oligoalginates (AOS) are promising biostimulants for wheat seedlings, particularly in improving root growth and increasing the ascorbate pool.

Future research should aim to clarify the molecular mechanisms underlying AOS activity, including their interactions with plant hormones and cell wall receptors. Field studies will be necessary to validate these effects under agricultural conditions and to evaluate their influence on yield and grain quality. In addition, optimizing production and application strategies, especially determining the most effective field dose, will be crucial for practical deployment. Furthermore, exploring how alginate-based biostimulants interact with the soil microbiome may reveal additional synergistic environmental benefits.

## Figures and Tables

**Table 1:** Mass characterization by size exclusion chromatography (SEC-MALLS) and ^1^HNMR spectroscopy analysis of alginates and the calculation of GulA and ManA. Corresponding to the SEC-MALLS, Mw corresponds to the molecular weight in Daltons and PDI the polydispersity index. For the ^1^HNMR, A_I_ correspond to the signal at 4.995 and 4.964 ppm, A_II_ the signal at 4.594 and 4.618 ppm and A_III_ the signal at 4.351 and 4.357 ppm. F_G_, F_M_, F_GG_, F_MM_, F_GM_ and M/G correspond to the guluronic fraction, mannuronic fraction, guluronic blocks, mannuronic blocks, alternative blocks of guluronic and mannuronic and the ratio mannuronic and guluronic acids, respectively.

Sample	SEC-MALLS		^1^H NMR							
	Mw (kDa)	PDI	AI	AII	AIII	$F_{G}$	$F_{M}$	$F_{GG}$	$F_{MM}$	M/G
*Alginate ratio M/G 1.4 HMW*	281.6	2.52	0.86	1.23	0.79	0.43	0.57	0.39	0.54	1.35
*Alginate ratio M/G 0.7 HMW*	234.3	2.41	2.73	3.12	1.45	0.60	0.40	0.32	0.12	0.67

**Table 2:** Seedlings length after 7 days of treatment with 1 g/L treatments compared with control (without treatment). Values are represented as mean values ± SE. Different letters show significant differences. Statistical differences were assessed using Kruskal–Wallis test with Dunn’s post hoc and BH correction for multiple comparison (*p*.adj ≤ 0.05).

Sample	Root Length	Shoot Length
**Control**	5.95 ± 0.22 ^c^	19.01 ± 0.23 ^a^
**Al M/G 0.7**	9.43 ± 0.43 ^a^	18.16 ± 0.33 ^a^
**Al M/G 1.4**	16.10 ± 0.84 ^b^	31.19 ± 1.34 ^b^
**Ca Al**	8.55 ± 0.31 ^a^	19.40 ± 0.25 ^a^

**Table 3:** Mass characterization of alginate and depolymerized products by thermic-acidic depolymerization by high performance size exclusion chromatography (SEC-MALLS). Thermic-acidic depolymerization fractions were obtained by filtration, filtrate is represented by AOS (mix of all the filtrates), and the retentate fraction designed by R followed by the number autoclave cycles.

Sample	Cycle	Mw (Da)	PDI	Uronic Acid Units	% Depolymerization
**Alginate**	0	281,000	2.52	1606	0
**Retentate**					
**R-1**	1	11,000	2.26	63	61
**R-2**	2	6970	1.61	40	97.5
**R-3**	3	4890	1.51	28	98.2
**R-4**	4	3560	1.49	20	98.7
**R-5**	5	3150	1.43	18	98.8
**R-6**	6	2570	1.37	15	99
**R-10**	10	1970	1.24	11	99.2
**Filtrate**					
**AOS**	1	586 ± 55	1.11 ± 0.08	3	/

**Table 4:** Filtrate mass obtained after each autoclave cycle and after ultrafiltration with a 3 kDa membrane.

**Cycle**	**Filtrate Mass (mg)**	**% Recovery**	**Cumulative % Recovery**
**1**	550.3	5.5	5.50
**2**	14.8	0.15	5.65
**3**	146.1	1.45	7.11
**4**	381	3.81	10.92
**5**	201	2.01	12.93
**6**	419	4.19	17.12
**7**	373.4	3.73	20.86
**8**	523.4	5.23	26.09
**9**	246	2.46	28.55
**10**	509.7	5.10	33.65
**Total**	3364.7	33.65	33.65

**Table 5:** ^1^H-NMR spectroscopy analysis of alginate and the thermic-acidic depolymerization retentates and the calculation of GulA and ManA. For the ^1^H-NMR, A I correspond to the signal at 4.995 and 4.964 ppm, A II the signal at 4.594 and 4.618 ppm and A III the signal at 4.351 and 4.357 ppm. FG, FM, FGG, FMM, FGM and M/G correspond to the guluronic fraction, mannuronic fraction, guluronic blocks, mannuronic blocks, alternative blocks of guluronic and mannuronic and the ratio mannuronic and guluronic acids, respectively. The retentate fraction is designed by R followed by the number autoclave cycles.

	**Signal**			**Fractions**				**Ratio**
**Sample**	**A_I_**	**A_II_**	**A_III_**	**F_G_**	**F_M_**	**F_GG_**	**F_MM_**	**M/G**
Al M/G 1.4	0.86	1.23	0.79	0.43	0.57	0.39	0.54	1.35
R 1	1	1.02	1.07	0.48	0.52	0.51	0.56	1.09
R 2	1	0.73	0.84	0.64	0.36	0.53	0.26	0.57
R 3	1	0.79	0.98	0.57	0.43	0.55	0.42	0.77
R 4	1	0.7	0.96	0.61	0.39	0.58	0.37	0.65
R 5	1	0.64	0.99	0.61	0.39	0.61	0.38	0.63
R 6	1	0.63	0.99	0.62	0.38	0.61	0.38	0.62
R 10	1	0.5	0.78	0.78	0.22	0.61	0.04	0.28

**Table 6:** Biomass increasement (normalized with control without treatment) of wheat seedlings submitted to alginate and depolymerized products at 50 mg/L and 500 mg/L. Values of fresh weight-roots (FWR), dry weight-roots (DWR), fresh weight-leaves (FWL) and dry weight-leaves (DWL) are represented as means ± SE.

		Roots			Leaves		
	Dose (mg/L)	% FW Increase	% DW Increase	Water Content (%)	% FW Increase	% DW Increase	Water Content (%)
*Al*	50	+24.6 ± 11.8	−0.6 ± 5.1	92.1 ± 0.4	+2.5 ± 0.3	+3.6 ± 3.1	87.9 ± 0.3
*R 1*	50	+15.2 ± 4.5	+27.9 ± 7.4	90.0 ± 0.3	+11.9 ± 6.7	+12.0 ± 5.1	88.1 ± 0.3
*R 3*	50	+4.6 ± 1.9	+17.3 ± 5.5	90.0 ± 0.6	+1.5 ± 2.7	+3.7 ± 2.5	88.1 ± 0.3
*R 10*	50	+19.1 ± 12.4	+24.4 ± 18.9	90.4 ± 0.4	−1.6 ± 1.4	−0.6 ± 5.2	89.3 ± 1.3
*AOS*	50	+7.1 ± 9.5	+16.8 ± 13.2	89.9 ± 0.3	+7.2 ± 4.1	+1.8 ± 4.8	88.8 ± 0.1
*Al*	500	30.1 ± 6.4	+27.8 ± 6.8	90.7 ± 0.3	+1.3 ± 3.7	+1.6 ± 2.8	88.1 ± 0.1
*R 1*	500	19.3 ± 3.7	+16.2 ± 5.4	91.3 ± 0.4	+5.0 ± 3.2	+2.5 ± 1.2	88.4 ± 0.4
*R 3*	500	16.4 ± 9.6	+16.3 ± 3.5	90.9 ± 0.5	+0.7 ± 6.4	−6.4 ± 8.4	89.0 ± 0.2
*R 10*	500	27.8 ± 13.1	+29.2 ± 9.0	90.6 ± 0.4	+11.8 ± 6.8	+4.9 ± 3.9	89.0 ± 0.4
*AOS*	500	17.9 ± 6.6	+31.2 ± 3.3	90.6 ± 0.4	+26.8 ± 1.8	+20.0 ± 5.5	89.0 ± 0.3

## Data Availability

The original contributions presented in this study are included in the article. Further inquiries can be directed to the corresponding author.

## Appendix A

**Table A1:** Biomass evaluation of wheat seedlings submitted to alginate and depolymerized products at 50 mg/L and 500 mg/L. Values of fresh weight-roots (FWR), dry weight-roots (DWR), fresh weight-leaves (FWL) and dry weight-leaves (DWL) are represented as means ± SE. Different letters show significant differences. Statistical differences were assessed using Kruskal–Wallis test with Dunn’s post hoc and BH correction for multiple comparison (*p*.adj ≤ 0.05).

		*Roots*		*Leaves*	
	Dose (mg/L^−1^)	FW (g)	DW (g)	FW (g)	DW (g)
*Control*	0	0.38 ± 0.021 ^a^	0.04 ± 0.002 ^ab^	1.34 ± 0.051 ^a^	0.16 ± 0.004 ^a^
*Al*	50	0.47 ± 0.029 ^a^	0.04 ± 0.001 ^a^	1.35 ± 0.030 ^a^	0.16 ± 0.005 ^a^
*R 1*	50	0.44 ± 0.020 ^a^	0.05 ± 0.002 ^ab^	1.44 ± 0.050 ^a^	0.17 ± 0.005 ^a^
*R 3*	50	0.43 ± 0.014 ^a^	0.04 ± 0.001 ^ab^	1.33 ± 0.030 ^a^	0.16 ± 0.002 ^a^
*R 10*	50	0.44 ± 0.033 ^a^	0.04 ± 0.004 ^ab^	1.31 ± 0.045 ^a^	0.14 ± 0.012 ^a^
*AOS*	50	0.41 ± 0.029 ^a^	0.04 ± 0.002 ^ab^	1.53 ± 0.031 ^a^	0.17 ± 0.004 ^a^
*Al*	500	0.50 ± 0.020 ^a^	0.05 ± 0.001 ^b^	1.23 ± 0.075 ^a^	0.15 ± 0.009 ^a^
*R 1*	500	0.47 ± 0.007 ^a^	0.04 ± 0.001 ^ab^	1.32 ± 0.030 ^a^	0.15 ± 0.003 ^a^
*R 3*	500	0.45 ± 0.020 ^a^	0.04 ± 0.002 ^ab^	1.35 ± 0.068 ^a^	0.15 ± 0.009 ^a^
*R 10*	500	0.47 ± 0.040 ^a^	0.05 ± 0.002 ^b^	1.44 ± 0.050 ^a^	0.16 ± 0.003 ^a^
*AOS*	500	0.48 ± 0.017 ^a^	0.05 ± 0.001 ^b^	1.54 ± 0.049 ^a^	0.17 ± 0.004 ^a^

**Table A2:** Primer pairs selected for q-PCR for PR1,2 and 3, PAL, CAT and one housekeeping gene TUB.

Sr. No.	Primer Pairs	Forward Sequence	Reverse Sequence	Genes These Primers Code for	Reference
**1**	PR1	5′-CATGCACCTTCGTATGCCTAACT-3′	5′-TGGCTAATTACGGCATTCCTTT-3′	Antifungal	[45,46,47]
**2**	PR2	5′-TCCTGGGTTCAGAACAATGTCC-3′	5′-TTGATGTTGACAGCCGGGTAGT-3′	β-1,3-glucanase	[45,46,47]
**3**	PR3	5′-GGGTGGACCTGCTGAACAAT-3′	5′-AGAACCATATCGCCGTCTTGA-3′	Chitinase (types I, II, IV, V, VI, VII)	[45,46,47]
**4**	PAL	5′-GTCGATTGAGCGTGAGATCAAC-3′	5′-CACGGGAGACGTCGATGAG-3′	Phenylalanine lyase	[45,46,47]
**5**	CAT	5′-TGCCTGTGTTTTTTATCCGAGA-3′	5′-CTGCTGATTAAGGTGTAGGTGTT-3′	Catalase	(Novel sequence—generated at a partner laboratory), [45,46,47]
**6**	TUB	5′-GGAGTACCCTGACCGAATGATG-3′	5′-AACGACGGTGTCTGAGACCTTT-3′	β-Tubulin (housekeeping gene)	[45,47,48]
